# Comprehensive analysis of the progression mechanisms of CRPC and its inhibitor discovery based on machine learning algorithms

**DOI:** 10.3389/fgene.2023.1184704

**Published:** 2023-07-05

**Authors:** Zhen Wang, Jing Zou, Le Zhang, Hongru Liu, Bei Jiang, Yi Liang, Yuzhe Zhang

**Affiliations:** ^1^ College of Basic Medical Sciences, Dali University, Dali, Yunnan, China; ^2^ The First Affiliated Hospital of Dali University, Dali, Yunnan, China; ^3^ Yunnan Key Laboratory of Screening and Research on Anti-pathogenic Plant Resources from West Yunnan (Cultivation), Dali, Yunnan, China; ^4^ Princess Margaret Cancer Centre, TMDT-MaRS Centre, University Health Network, Toronto, ON, Canada

**Keywords:** castration-resistant prostate cancer (CRPC), weighted gene coexpression network analysis (WGCNA), machine learning algorithm, CCNA2, Cks2, virtual screening, Aprepitant, Dolutegravir

## Abstract

**Background:** Almost all patients treated with androgen deprivation therapy (ADT) eventually develop castration-resistant prostate cancer (CRPC). Our research aims to elucidate the potential biomarkers and molecular mechanisms that underlie the transformation of primary prostate cancer into CRPC.

**Methods:** We collected three microarray datasets (GSE32269, GSE74367, and GSE66187) from the Gene Expression Omnibus (GEO) database for CRPC. Differentially expressed genes (DEGs) in CRPC were identified for further analyses, including Gene Ontology (GO), Kyoto Encyclopedia of Genes and Genomes (KEGG), and gene set enrichment analysis (GSEA). Weighted gene coexpression network analysis (WGCNA) and two machine learning algorithms were employed to identify potential biomarkers for CRPC. The diagnostic efficiency of the selected biomarkers was evaluated based on gene expression level and receiver operating characteristic (ROC) curve analyses. We conducted virtual screening of drugs using AutoDock Vina. *In vitro* experiments were performed using the Cell Counting Kit-8 (CCK-8) assay to evaluate the inhibitory effects of the drugs on CRPC cell viability. Scratch and transwell invasion assays were employed to assess the effects of the drugs on the migration and invasion abilities of prostate cancer cells.

**Results:** Overall, a total of 719 DEGs, consisting of 513 upregulated and 206 downregulated genes, were identified. The biological functional enrichment analysis indicated that DEGs were mainly enriched in pathways related to the cell cycle and metabolism. CCNA2 and CKS2 were identified as promising biomarkers using a combination of WGCNA, LASSO logistic regression, SVM-RFE, and Venn diagram analyses. These potential biomarkers were further validated and exhibited a strong predictive ability. The results of the virtual screening revealed Aprepitant and Dolutegravir as the optimal targeted drugs for CCNA2 and CKS2, respectively. *In vitro* experiments demonstrated that both Aprepitant and Dolutegravir exerted significant inhibitory effects on CRPC cells (*p* < 0.05), with Aprepitant displaying a superior inhibitory effect compared to Dolutegravir.

**Discussion:** The expression of CCNA2 and CKS2 increases with the progression of prostate cancer, which may be one of the driving factors for the progression of prostate cancer and can serve as diagnostic biomarkers and therapeutic targets for CRPC. Additionally, Aprepitant and Dolutegravir show potential as anti-tumor drugs for CRPC.

## Introduction

Prostate cancer is the second most common cancer in men after lung cancer, affecting millions of men worldwide, accounting for 7% of newly diagnosed cancers in men worldwide and 15% in developed regions, and is one of the leading causes of cancer-related deaths among men (Rebello et al., 2021). Most patients are initially diagnosed with primary prostate cancer, and the 5-year survival rate is over 95%. In contrast, for approximately 5% of men with distant metastatic prostate cancer, the estimated 5-year survival rate is only 28% (Siegel et al., 2019). Androgen deprivation therapy (ADT) is the primary treatment option for patients with metastatic disease and recurrent prostate cancer after local treatment. Patients’ initial response to castration therapy is often very favourable, accompanied by a significant improvement in clinical symptoms and a rapid resolution of biochemical reactions (Harris et al., 2009; Marques et al., 2010). However, almost all patients treated with ADT eventually develop castration-resistant prostate cancer (CRPC), as evidenced by imaging progression or increased prostate-specific antigen (PSA) despite castration levels of testosterone (Davies et al., 2019). CRPC has a poor prognosis with an average survival time of only 16–18 months from progression (Sun et al., 2010). Initially, CRPC was only treated with a limited regimen, such as docetaxel chemotherapy. After 2010, docetaxel, rivastigmine, and abiraterone acetate provided patients with new options to prolong the survival time of CRPC (Tannock et al., 2004; Kantoff et al., 2010; Pal et al., 2010; Paller and Antonarakis, 2011). To date, the treatment of CRPC remains a major clinical challenge. Rational combination therapy, genome-driven therapy, and immunotherapy for patients are being tested for this disease. Currently, there is a critical need to define molecular biomarkers to select patients for treatment. Exploring the molecular markers between primary prostate cancer and CRPC will help diagnose the disease, better understand the mechanism of primary prostate cancer transforming to CRPC, and provide new targets for treating CRPC.

Weighted gene coexpression network analysis (WGCNA) is an algorithm used to process gene expression correlations for microarray data (Langfelder and Horvath, 2008). WGCNA found clusters of highly related genes for clustering and associated modules with phenotypes to obtain the modules most related to phenotypic traits and finally obtained the hub genes in these modules. Machine learning algorithms have shown great promise in studying the potential relationships of high-dimensional data (Tshitoyan et al., 2019). Recently, machine learning has been increasingly used to analyse high-dimensional transcriptome data and identify biologically significant feature genes, with promising results (Bogard et al., 2019; Kachroo et al., 2019; Huang et al., 2020). However, these techniques have not been applied to identify potential biomarkers of CRPC.

In this study, we investigated the differentially expressed genes (DEGs) between primary prostate cancer and CRPC and conducted several functional enrichment analyses, including Gene Ontology (GO), Kyoto Encyclopedia of Genes and Genomes (KEGG), Disease Ontology (DO), and Gene set enrichment analysis (GSEA). Moreover, we utilized WGCNA and machine learning algorithms such as Least Absolute Shrinkage and Selection Operator (LASSO) logistic regression and Support Vector Machine-Recursive Feature Elimination (SVM-RFE) to identify potential biomarkers of CRPC. We also evaluated the variation in immune cell infiltration between primary prostate cancer and CRPC using the single-sample gene set enrichment analysis (ssGSEA) algorithm, and investigated the correlation between the identified biomarkers and immune cell infiltration. Finally, we employed virtual screening methods to discover potential antagonists that bind to the predicted active site, and conducted preliminary studies on the mechanisms of action of the drugs through *in vitro* experiments. In summary, these results help us to understand the molecular mechanisms underlying the progression of primary prostate cancer to CRPC and further explore potential drugs for the treatment of CRPC.

## Materials and methods

### Collection and processing of microarray data

Three microarray datasets of CRPC (GSE32269, GSE74367, and GSE66187) were collected from the Gene Expression Omnibus (GEO) database (http://www.ncbi.nlm.nih.gov/geo/). GSE32269 was based on the platforms of GPL96 (Cai et al., 2013), GSE74367 was based on the platforms of GPL15659 (Roudier et al., 2016), and GSE66187 was based on the platforms of GPL15659 (Zhang et al., 2015). We combined the GSE32269 and GSE74367 datasets, which included 33 primary prostate cancer samples and 74 CRPC samples, into the training cohort and the GSE66187 dataset, which included 24 primary prostate cancer samples and 71 CRPC samples, into the testing cohort (Table 1). All data were batch eliminated by the Surrogate Variable Analysis (SVA) algorithm (Parker et al., 2014).

**TABLE 1:** Microarray data details in this study.

GEO series	Primary prostate cancer	CRPC	Data type
GSE32269	22	29	Training
GSE74367	11	45	Training
GSE66187	24	71	Testing

GEO: gene expression omnibus; CRPC: castration-resistant prostate cancer.

### Identification of DEGs

Using the “limma” R package (Ritchie et al., 2015), the DEGs between CRPC and primary prostate cancer in the training cohort were detected (|log2fold change (FC)| > 2, and *p* < 0.001). Subsequently, GO enrichment analysis was performed to investigate the biological process (BP), molecular functions (MF), and cellular components (CC). KEGG enrichment analysis was used to explore the signalling pathways associated with DEGs. The biological enrichment for GO and KEGG analyses was achieved using the “Cluster Profiler” R package (*p* < 0.05, FDR<0.05). Finally, GSEA was performed to investigate the DEG-enriched biological pathways using an ordered gene expression matrix based on Pearson correlation between genes.

### WGCNA

We created a weighted gene coexpression network through the expression matrix of DEGs to screen potential genes related to CRPC based on the “WGCNA” R package (Langfelder and Horvath, 2008). First, we clustered all samples, deleted the samples with low correlation, and calculated the Pearson correlation coefficient between each pair of genes to evaluate the expression similarity of genes and obtain the correlation matrix. Subsequently, we transformed the adjacency matrix into a topological overlap matrix (TOM) and performed hierarchical clustering to identify the modules and calculate the feature genes. We then represented the different branches of a gene module with a similar expression profile with an appropriate colour, with the minimum number of genes in the module set to 60. Finally, the genes in the modules with the highest correlation were screened with gene importance > 0.5 and gene-module correlation > 0.8 as the threshold, and the genes screened were considered vital genes.

### Recognition of potential biomarkers in CRPC based on the machine learning algorithm

The LASSO logistic regression algorithm (Tibshirani, 1996) was used to eliminate overfitting in modules based on the “glmnet” R Package. SVM-RFE is an efficient feature selection algorithm that iteratively removes the features with the lowest weights. In each iteration, the current SVM-RFE model is evaluated by k-fold cross-validation. Finally, the classifier model with the highest precision is constructed, and the optimal variable is found (Lin et al., 2012). The execution of the SVM-RFE algorithm was based on the “e1071” R packet (Huang et al., 2014). Ultimately, the intersecting genes in the two machine learning algorithms were considered diagnostic biomarkers.

### Evaluation of the effects of diagnostic biomarkers

We assessed the ability of diagnostic biomarkers to differentiate primary prostate cancer from CRPC through gene expression and ROC curves. Boxplots were used to demonstrate gene expression, and *p* < 0.05 indicated significant differences in gene expression. A receiver operating characteristic (ROC) curve was generated via the area under the ROC curve (AUC) value to estimate the predictive utility of identified biomarkers based on the “pROC” package. The testing cohort data further verified the difference in biomarker expression and prediction reliability.

### Evaluation of immune cell infiltration

We evaluated the infiltration of 28 immune cells in primary prostate cancer and CRPC and presented them with a heatmap and violin plot based on ssGSEA. In addition, to explore the immune-related mechanism of the biomarkers identified in CRPC transformation, we further evaluated the correlation between genes and immune cell infiltration.

### Virtual screening and molecular docking of drugs

We retrieved protein crystal structure files from the Protein Data Bank (PDB) database (https://www.rcsb.org/) and processed protein molecules by removing water molecules and ligand files using the pymol software. Subsequently, we used the getbox plugin to obtain the active pockets from the protein structures and performed hydrogenation of protein files in AutoDocktools software. The protein files were then converted to pdbqt file format for subsequent virtual screening. We downloaded US Food and Drug Administration (FDA) approved small molecule drugs (fda+for+sale) from the ZINC15 database (https://zinc15.docking.org/) and split and converted the files using Open Babel. We conducted virtual screening of small molecule drugs using AutoDock vina, docking each drug molecule with the protein five times (Trott and Olson, 2010). Finally, the online tool Protein-Ligand Interaction Profiler (PLIP) (https://plip-tool.biotec.tu-dresden.de/plip-web/plip/index) was employed to analyze the interaction between the top 8 ligands with the strongest affinity and proteins (Adasme et al., 2021). The results were further visualized using pymol and ligplot software. Based on the combined analysis of affinity and interaction, the small molecule ligand with the highest binding capacity to the receptor protein was selected.

### Cell culture and drug treatment

In this study, DU145 and PC-3 cells were obtained from the Chinese Cell Bank of the Shanghai Institute of Biochemistry and Cell Biology (https://www.cellbank.org.cn/). Cells were cultured in DMEM medium (Gibco, Life Technologies, China) supplemented with 10% fetal bovine serum (SERANA, Europe) and 1% penicillin-streptomycin (Beyotime, China). The cells were seeded in culture flasks (Nest, China) and maintained in a humidified incubator at 37°C and 5% CO_2_. Aprepitant was purchased from aladdin-ShangHai-China and Dolutegravir was purchased from Macklin-ShangHai-China. The stock solutions of these drugs were prepared in sterile dimethyl sulfoxide (DMSO), with a final concentration of DMSO in the medium below 0.1% of the total volume.

### Cell viability assay

DU145 and PC-3 cells were seeded at concentrations of 8 × 10^3^ and 4 × 10^3^ cells/well in 96-well plates, with 100 μL complete culture medium per well, and incubated for 24 h at 37°C in a humidified incubator. Aprepitant concentrations were set at 0 μM (untreated group), 5 μM, 10 μM, 15 μM, 20 μM, 30 μM, and 40 μM, while Dolutegravir concentrations were set at 0 μM (untreated group), 10 μM, 20 μM, 40 μM, 80 μM, and 100 μM, and cells were incubated for 24, 48, and 72 h. After drug incubation, 10 μL of Cell Counting Kit-8 (CCK-8) solution (Bioss, China) was added to each well, and the plates were incubated at 37°C for 1 h. The absorbance at 450 nm was measured using an enzyme-linked immunosorbent assay (ELISA) reader.

### Wound migration assay

DU145 cells were seeded in a 6-well culture plate at a concentration of 2 × 10^6^ cells/well and incubated overnight. When the cell confluence reached 90%, the culture medium was removed, and a scratch was made using a 200 μL pipette tip. The cells were washed with Phosphate-buffered saline (PBS) and images were captured at a fixed position using a microscope (X100). The culture medium was then replaced with serum-free medium (control group) or serum-free medium containing Aprepitant (20 μM) and Dolutegravir (80 μM) (treatment group). The cells were incubated at 37°C for 24 h in a cell culture incubator before images were captured again. The area of the scratch was calculated using ImageJ software.

### Cell invasion assay

For the transwell invasion assay, we used 24-well transwell plates with an 8 mm pore size (Corning, United States). The polycarbonate membrane was coated with 8.1 mg/mL Matrigel matrix (Solarbio, Beijing, China) and incubated at 37°C for 2 h to solidify the Matrigel matrix. 2 × 10^4^ cells were seeded in the upper chamber containing 200 µL of serum-free medium, while 600 µL of medium containing 10% FBS was added to the lower chamber. After incubation at 37°C for 24 h in a cell culture incubator, the invasive cells were fixed with 4% paraformaldehyde and stained with 0.1% crystal violet. Images of 5 randomly selected fields were captured using a microscope (X200), and the cells were counted using ImageJ software.

### Statistical analysis

All statistical analyses were performed using R software (version 4.1.2). A t-test was used for comparisons between two groups, while a one-way ANOVA test was used for comparisons involving three or more groups. Statistical significance was con-sidered for *p < 0.05, **p < 0.01, and ***p < 0.001.

## Results

### Identification of differentially expressed genes between primary prostate cancer and CRPC

**FIGURE 1:**
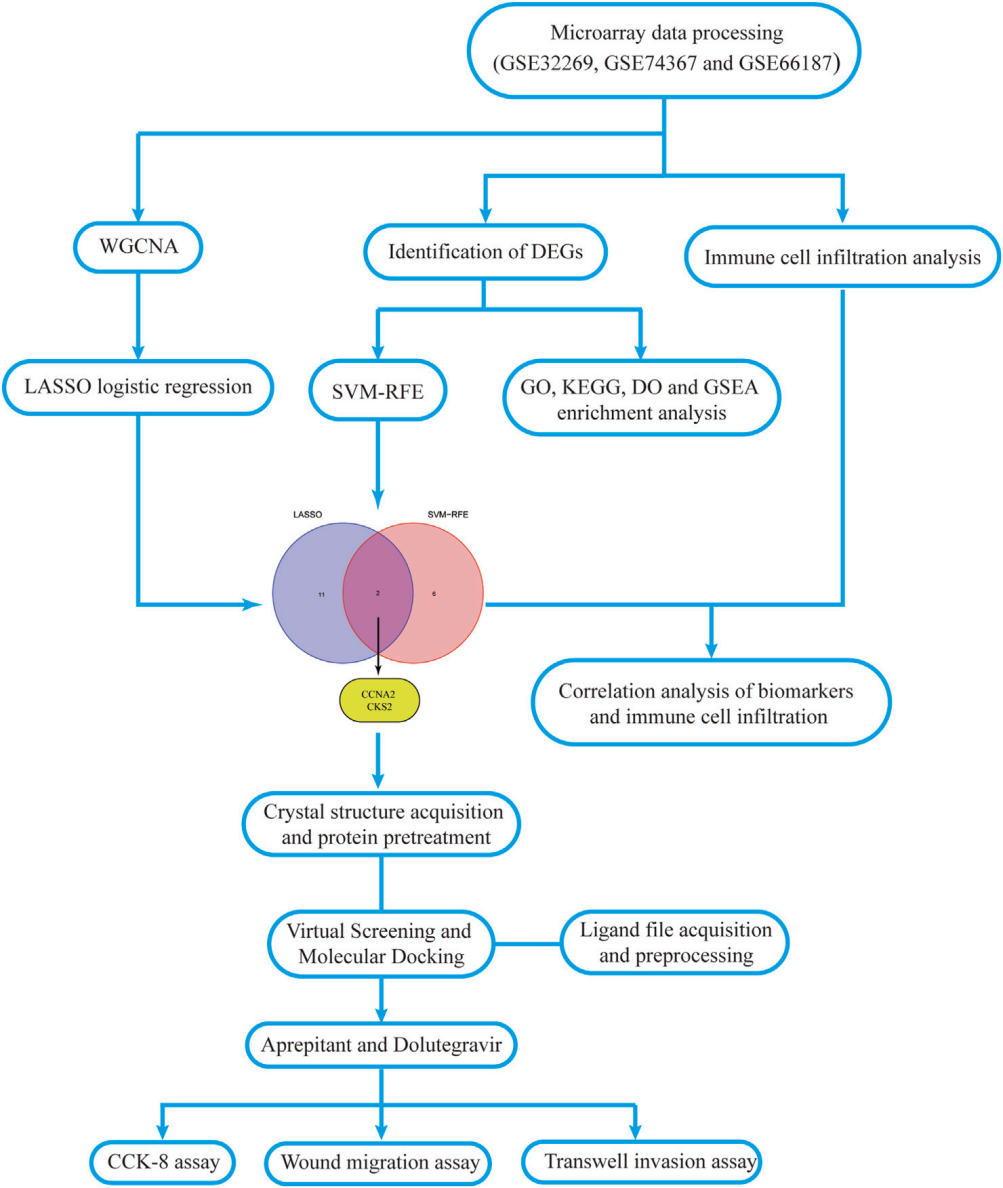
Workflow diagram of this study.

**FIGURE 2:**
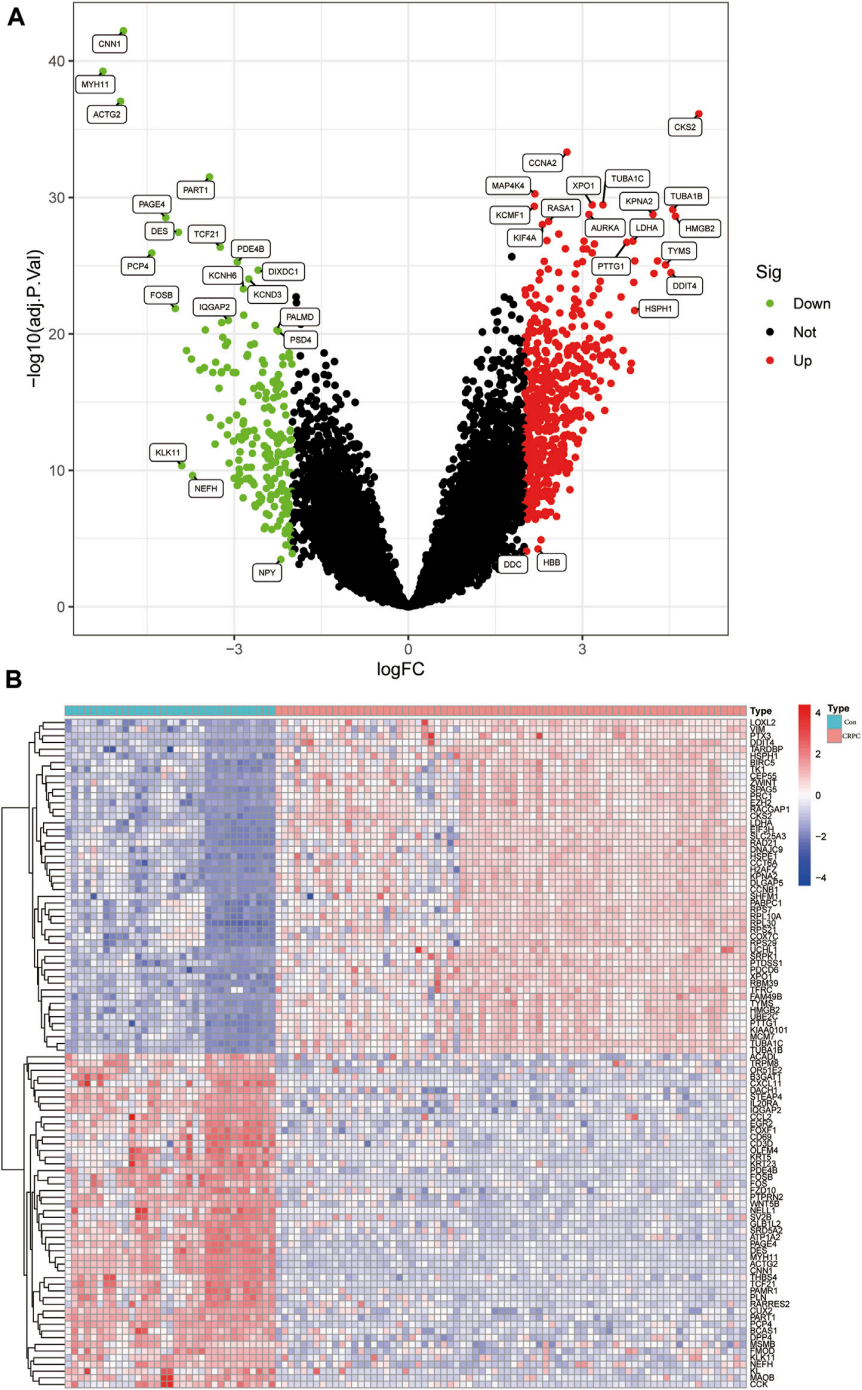
Identification of DEGs in the CRPC training cohort. **(A)** Volcano plot of the DEGs. **(B)** Heatmap of DEGs in the CRPC training cohort.

The flowchart of this study is shown in Figure 1. The detailed information of the sample quantity in the microarray data is shown in Table 1. By comparing CRPC with primary prostate cancer samples, we identified a total of 719 DEGs, including 513 upregulated genes and 206 downregulated genes (|log2FC|>2, *p* < 0.05), displayed in the volcano plot and heatmap (Figures 2A,B).

### GO, KEGG, and GSEA enrichment analyses

**FIGURE 3:**
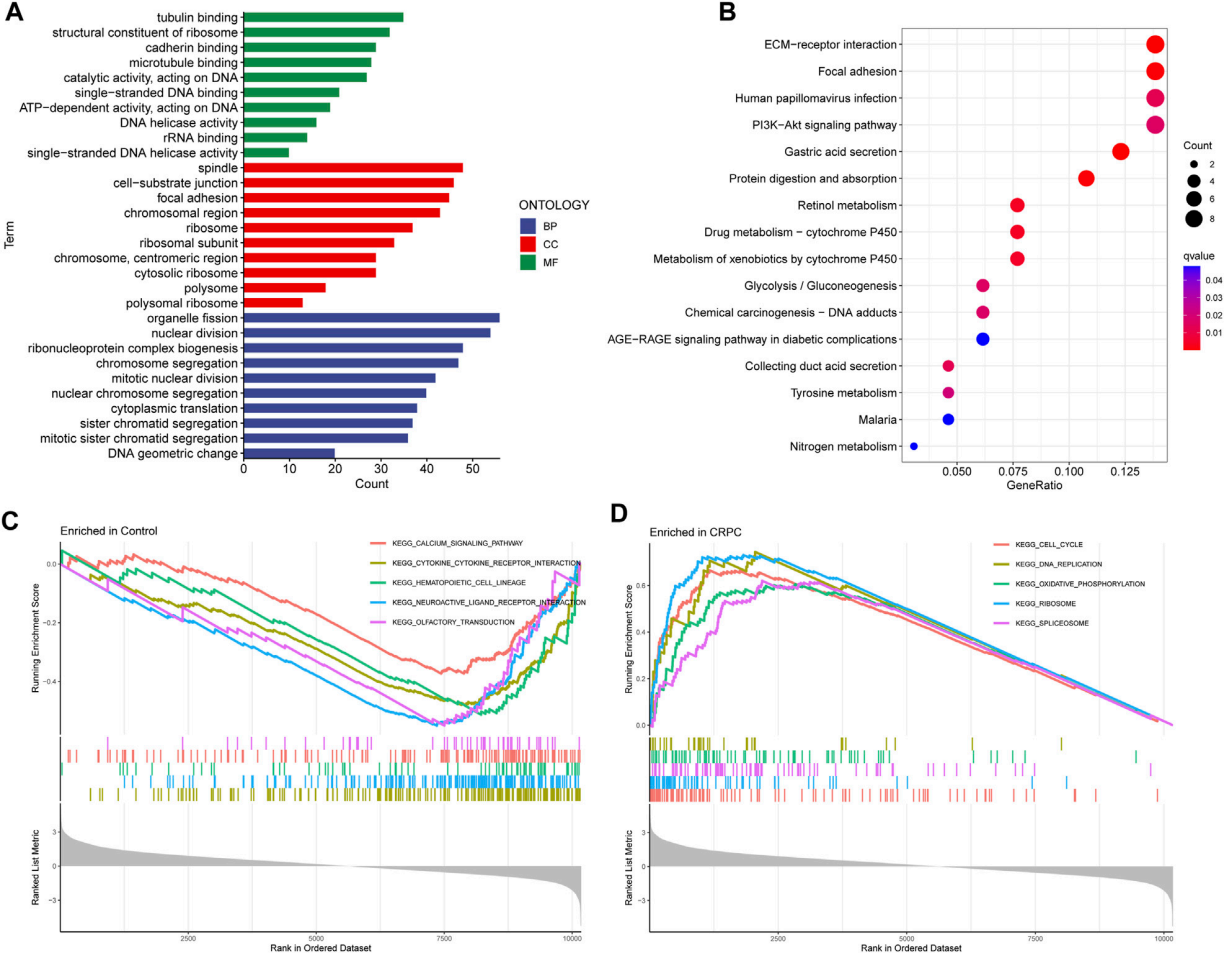
GO, KEGG, and GSEA enrichment analyses. **(A)** GO enrichment analysis. **(B)** KEGG pathway enrichment analysis. **(C, D)** Five enriched signalling pathways in primary prostate cancer and CRPC samples.

Enrichment analyses of GO, KEGG, and GSEA were performed to study the biological function of DEGs. The GO analysis indicated that these DEGs were mainly involved in biological processes associated with organelle fission, spindles, and tubulin binding (Figure 3A; Supplementary Table S1). In addition, the KEGG enrichment analysis indicated that DEGs were mainly enriched in the signalling pathways related to ECM Receptor Interaction and Focal adhesion (Figure 3B; Supplementary Table S2). Finally, the GSEA results showed that signalling pathway-related pathways were enriched in primary prostate cancer samples, while cell cycle-related signalling pathways were enriched in CRPC samples (Figures 3C,D; Supplementary Table S3).

### WGCNA

**FIGURE 4:**
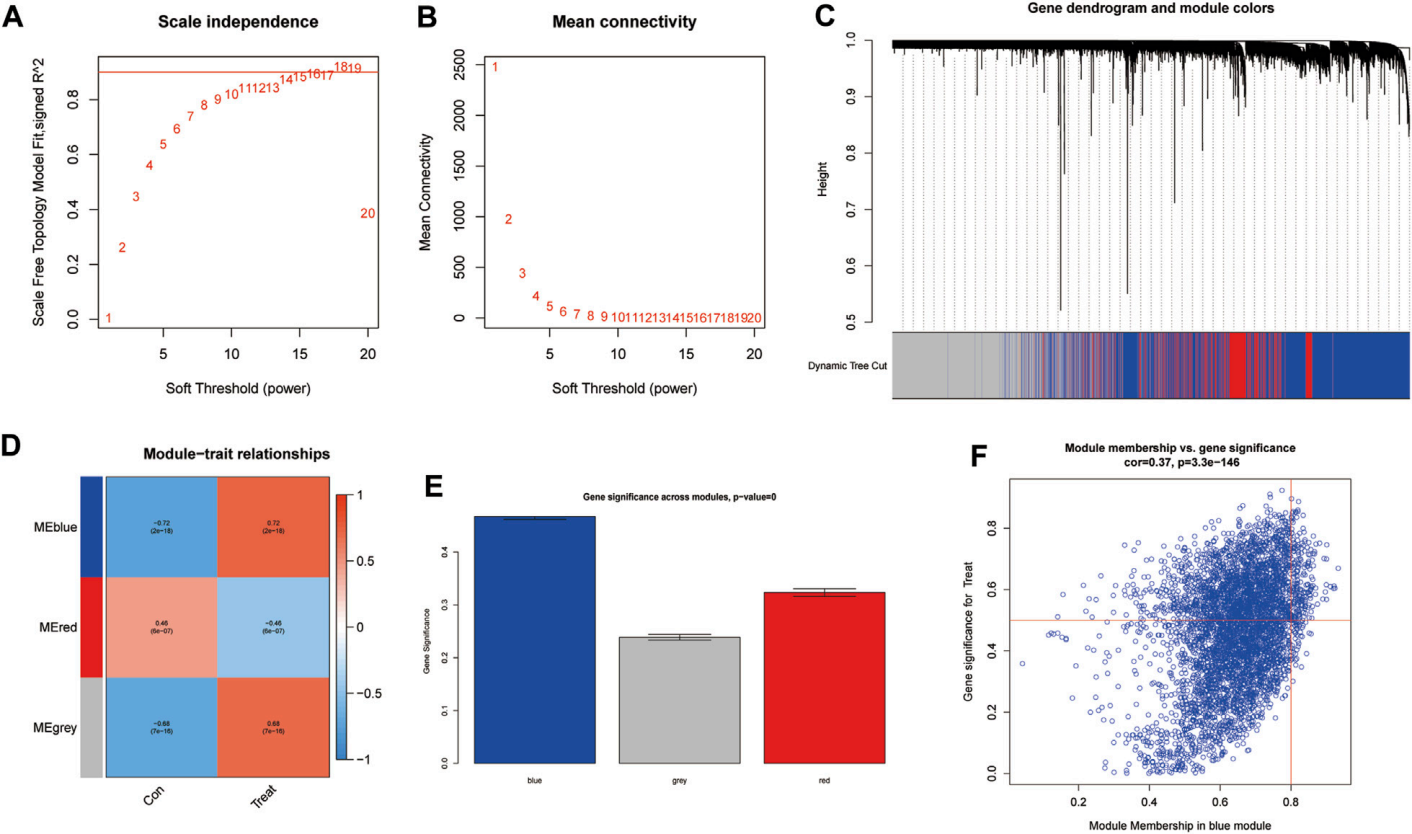
Identification of significant gene modules correlated with CRPC with WGCNA. **(A,B)** Analysis of the network topology for various soft-threshold powers. **(C)** Cluster dendrogram of representative gene modules. **(D)** Correlation of each ME with CRPC. **(E)** The importance of module genes in CRPC. **(F)** Screening of key genes in the module with criteria (importance > 0.5, correlation > 0.8).

To further identify critical genes in CRPC, WGCNA was performed using 719 DEGs. Cluster analysis of the samples showed correlations among all samples, and the expression matrix of DEGs in all 107 samples was used to construct a weighted gene coexpression network. A soft threshold of 14 (R2 = 0.86) was set to construct the scale-free network (Figures 4A,B). In addition, by combining the modules with high correlation, three coexpression modules were screened out in the weighted gene coexpression network, and the minimum number of genes was set to 60 (Figure 4C). Then, we calculated the Pearson correlation between the module eigengene (ME) and CRPC for each module. The blue module had the highest correlation and significance with CRPC (cor = 0.72, P = 2e-18) and was selected as the target module (Figures 4D,E). Finally, we obtained 219 target genes for subsequent analysis by gene importance and module correlation as screening criteria (importance > 0.5, correlation > 0.8) (Figure 4F).

### Identification of potential biomarkers of CRPC by machine learning algorithms

**FIGURE 5:**
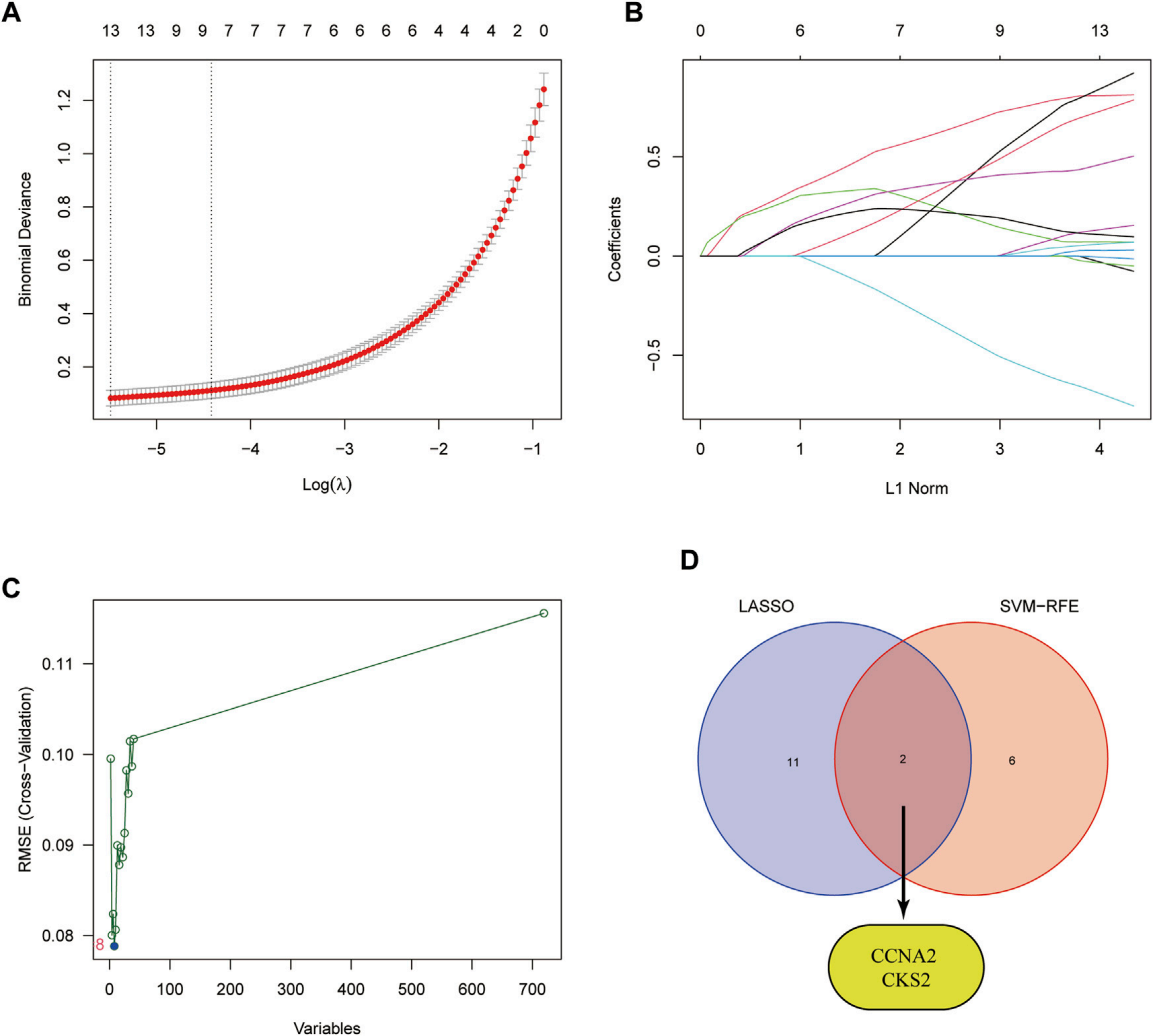
Identification of diagnostic biomarkers. **(A)** Cross-validation for tuning the parameter selection in the LASSO regression. **(B)** LASSO regression of the 13 module genes. **(C)** SVM-RFE algorithm. **(D)** Identification of intersecting genes from the two machine learning algorithms by Venn diagram.

To further identify potential biomarkers of CRPC from target genes, we used two machine learning algorithms, LASSO logistic regression and SVM-RFE. First, we screened the overfitted genes in the modular genes using the LASSO regression algorithm, of which 13 genes (AZIN1, CCNA2, CKS2, COPS5, FXR1, HNRNPA1, HSPD1, LDHA, MRPL13, NDUFAB1, PAGE4, RASA1, TUBA1C) were identified as potential diagnostic biomarkers (Figures 5A,B). Subsequently, through the SVM-RFE algorithm, we obtained 8 genes (CKS2, PART1, CNN1, KCMF1, MAP4K4, XPO1, PTTG1, and CCNA2) from DEGs as potential diagnostic biomarkers based on the training cohort data (Figure 5C). Finally, we selected the intersecting genes of the two gene sets through the Venn diagram and obtained two genes (CCNA2 and CKS2) as the final diagnostic biomarkers (Figure 5D).

### Assessment of the expression levels and diagnostic capabilities of potential biomarkers

**FIGURE 6:**
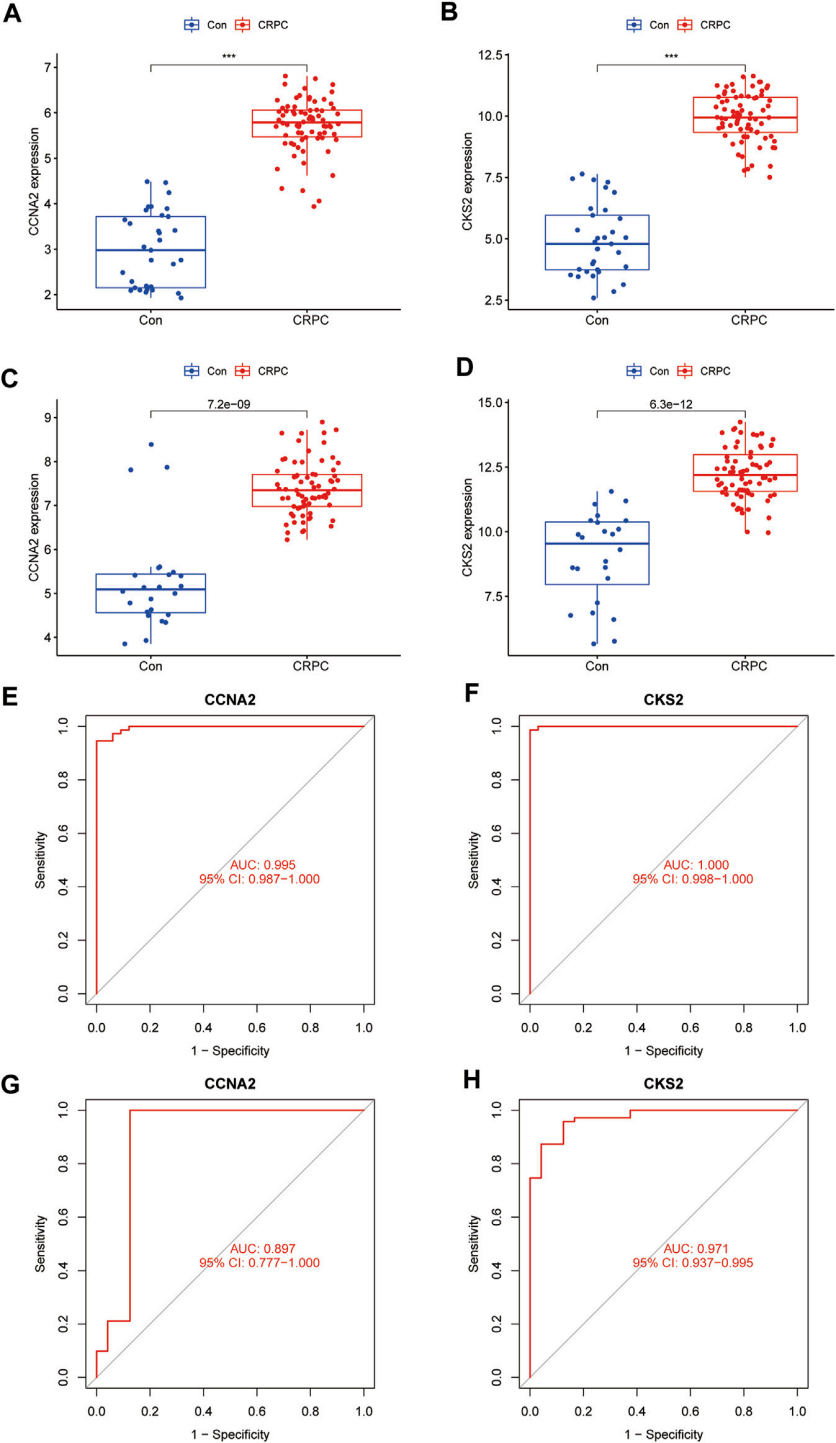
Verification of the identified biomarkers. **(A–D)** Box plots for the differential expression analysis in the CRPC training and testing cohorts. **(E–H)** ROC curves evaluate the diagnostic ability in the CRPC training and testing cohorts.

The expression of CCNA2 and CKS2 in the training cohort CRPC samples was significantly higher than that in the primary prostate cancer samples (*p* < 0.001) (Figures 6A,B). The same results were obtained for validation with testing cohort data (Figures 6C,D). To evaluate the predictive performance of diagnostic biomarkers, ROC analysis was performed. The AUC values of CCNA2 and CKS2 in the training cohort were found to be 0.995 and 1.000, respectively, showing reliable predictive power (Figures 6E,F). Meanwhile, the AUC values of CCNA2 and CKS2 in the testing cohort were 0.897 and 0.971, respectively, and these results indicated that CCNA2 and CKS2 are efficient diagnostic biomarkers (Figures 6G,H).

### Immune cell infiltration analysis

**FIGURE 7:**
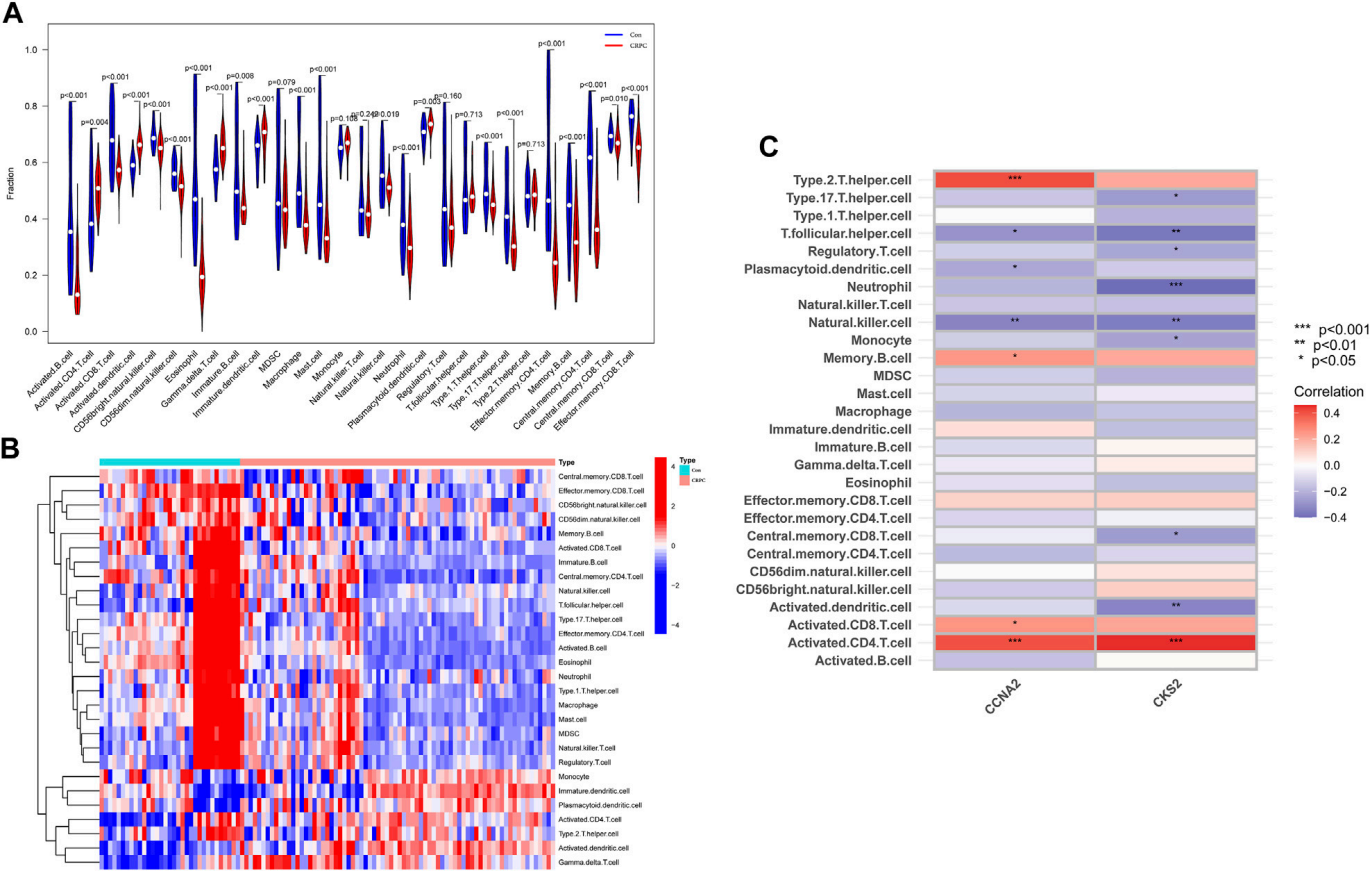
Immune cell infiltration analysis. **(A,B)** Heatmap and volcano plot for immune cell infiltration analysis. **(C)** Correlation of the identified biomarkers CCNA2 and CKS2 with immune cell infiltration.

With the ssGSEA method, we further analysed the difference in immune cell infiltration between primary prostate cancer and CRPC and explored the correlation between diagnostic biomarkers and immune cell infiltration. Most immune cell infiltration was significantly different in primary prostate cancer and CRPC, and most immunocytes were more infiltrated in primary prostate cancer than in CRPC (Figures 7A,B). Correlation analysis showed a negative correlation between CKS2 and most immune cells, while both CCNA2 and CKS2 were negatively correlated with NK cell infiltration (Figure 7C).

### Virtual screening

**FIGURE 8:**
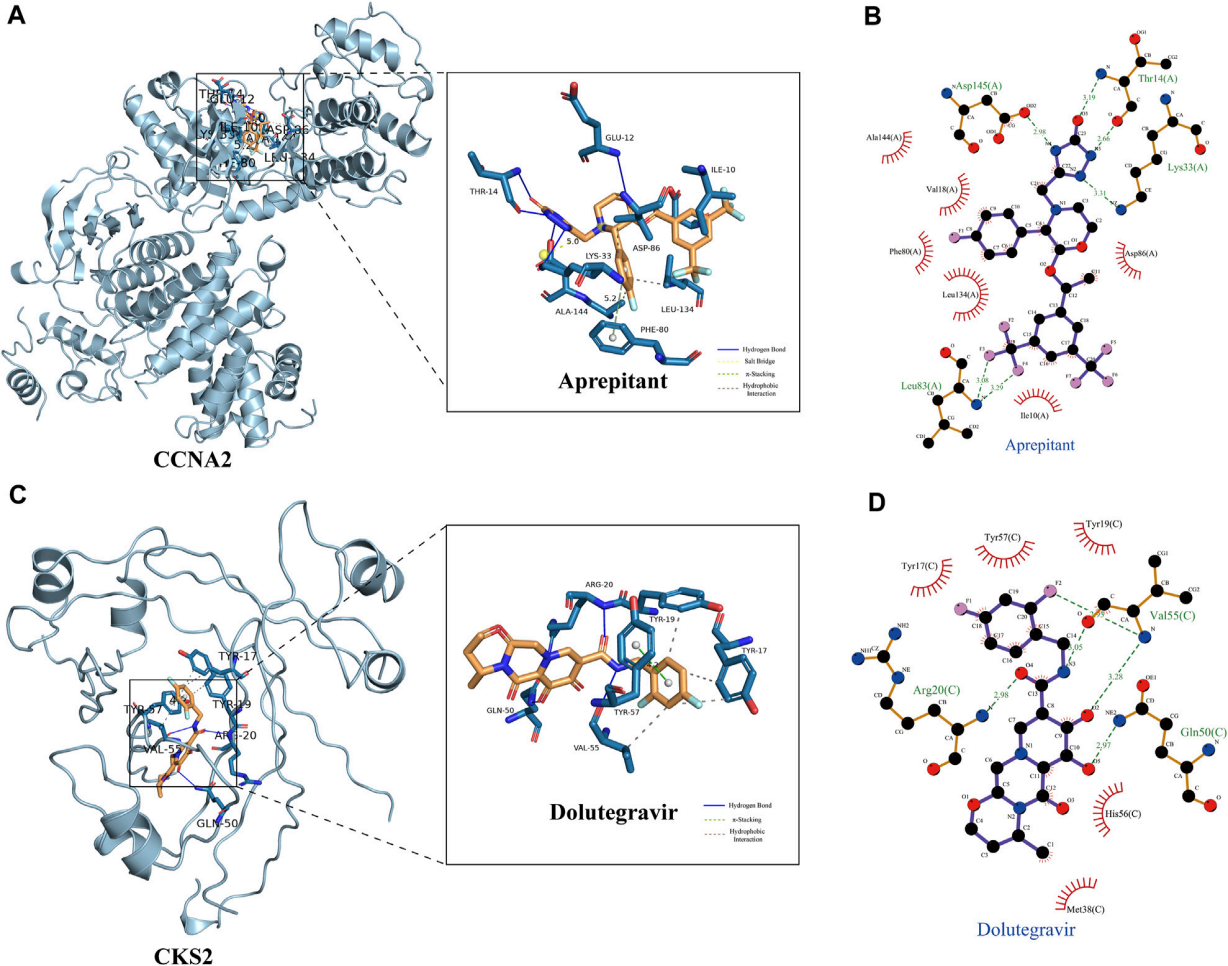
Demonstration of Protein-Ligand Interaction. **(A)** Three-dimensional display of CCNA2 crystal structure and its interaction with Aprepitant. **(B)** Two-dimensional display of the interaction between CCNA2 and Aprepitant. **(C)** Three-dimensional display of CKS2 crystal structure and its interaction with Dolutegravir. **(D)** Two-dimensional display of the interaction between CKS2 and Dolutegravir.

Based on our screening results, the top 8 drug molecules with the strongest binding affinity to CCNA2 were identified as Differin, Dihydroergotamine, Ergotamine, Dutasteride, Coreg, Aprepitant, Nilotinib, and Abiraterone. The affinity between Aprepitant and CCNA2 was −11.0 kcal/mol. Aprepitant interacted with CCNA2 protein through one π-stacking, one salt bridge, and six hydrogen bonds, demonstrating strong binding capacity (Figures 8A,B). In addition, we also identified the top 8 drugs with the lowest binding energy for CKS2, namely, Ergotamine, Indocyanine Green, Dolutegravir, Dihydroergotamine, Midostaurin, Glimepiride, Trypan Blue free acid, and Dutasteride. Among these, the affinity of Dolutegravir was −12.0 kcal/mol, and it interacted with CKS2 through one π-stacking and five hydrogen bonds, demonstrating strong binding capacity. (Figures 8C,D). Based on a combination of our virtual screening and molecular docking results, we selected drugs with the strongest protein affinity for further experiments, as listed in the table below (Table 2). It is noteworthy that although Trypan Blue free acid showed strong affinity to CKS2, it is a hydrophilic compound that is not membrane-permeable, meaning it cannot easily cross the lipid bilayer of the cell membrane to react inside the cell. Therefore, it is not suitable as our experimental object. Consequently, we will employ Aprepitant and Dolutegravir for further experimentation.

**TABLE 2:** The affinity and interaction of the top 8 compounds from virtual screening results.

Target	Number	Ligand	Chemical formula	Pubchem number	Zinc number	Affinity (kcal/mol)	Noncovalent interactions
CCNA2	1	Differin	C28H28O3	60164	ZINC000003784182	−11.8	0
	2	Dihydroergotamine	C33H37N5O5	10531	ZINC000003978005	−11.4	1 π-Cation, 2 H-bond
	3	Ergotamine	C33H35N5O5	8,223	ZINC000052955754	−11.1	1 π-Cation, 5 H-bond
	4	Dutasteride	C27H30F6N2O2	6918296	ZINC000003932831	−11.1	0
	5	Coreg	C24H26N2O4	185395	ZINC000001530579	−11.0	3 H-bond
	6	Aprepitant	C23H21F7N4O3	135413536	ZINC000027428713	−11.0	1 π-Stacking, 1 Salt Bridge, 6 H-bond
	7	Nilotinib	C28H22F3N7O	644241	ZINC000006716957	−10.9	1 Halogen Bond, 2 H-bond
	8	Abiraterone	C24H31NO	132971	ZINC000003797541	−10.8	4 H-bond
CKS2	1	Ergotamine	C33H35N5O5	8,223	ZINC000052955754	−12.3	2 π-Stacking, 1 Salt Bridge
	2	Indocyanine Green	C43H49N2O6S2^+^	19191	ZINC000008101127	−12.1	1 π-Cation, 2 Salt Bridge
	3	Dolutegravir	C20H19F2N3O5	54726191	ZINC000058581064	−12.0	1 π-Stacking, 5 H-bond
	4	Dihydroergotamine	C33H37N5O5	10531	ZINC000003978005	−11.8	3 π-Stacking
	5	Midostaurin	C35H30N4O4	9829523	ZINC000100013130	−11.7	3 π-Cation, 2 H-bond
	6	Glimepiride	C24H34N4O5S	3,476	ZINC000100070954	−11.5	1 π-Stacking, 2 H-bond
	7	Trypan Blue free acid	C34H28N6O14S4	6,297	ZINC000169289767	−11.4	1 π-Cation, 2 Salt Bridge, 7 H-bond
	8	Dutasteride	C27H30F6N2O2	6918296	ZINC000003932831	−11.2	1 Halogen Bonds, 2 H-bond

### Effects of Aprepitant and Dolutegravir on the viability of CRPC cells

**FIGURE 9:**
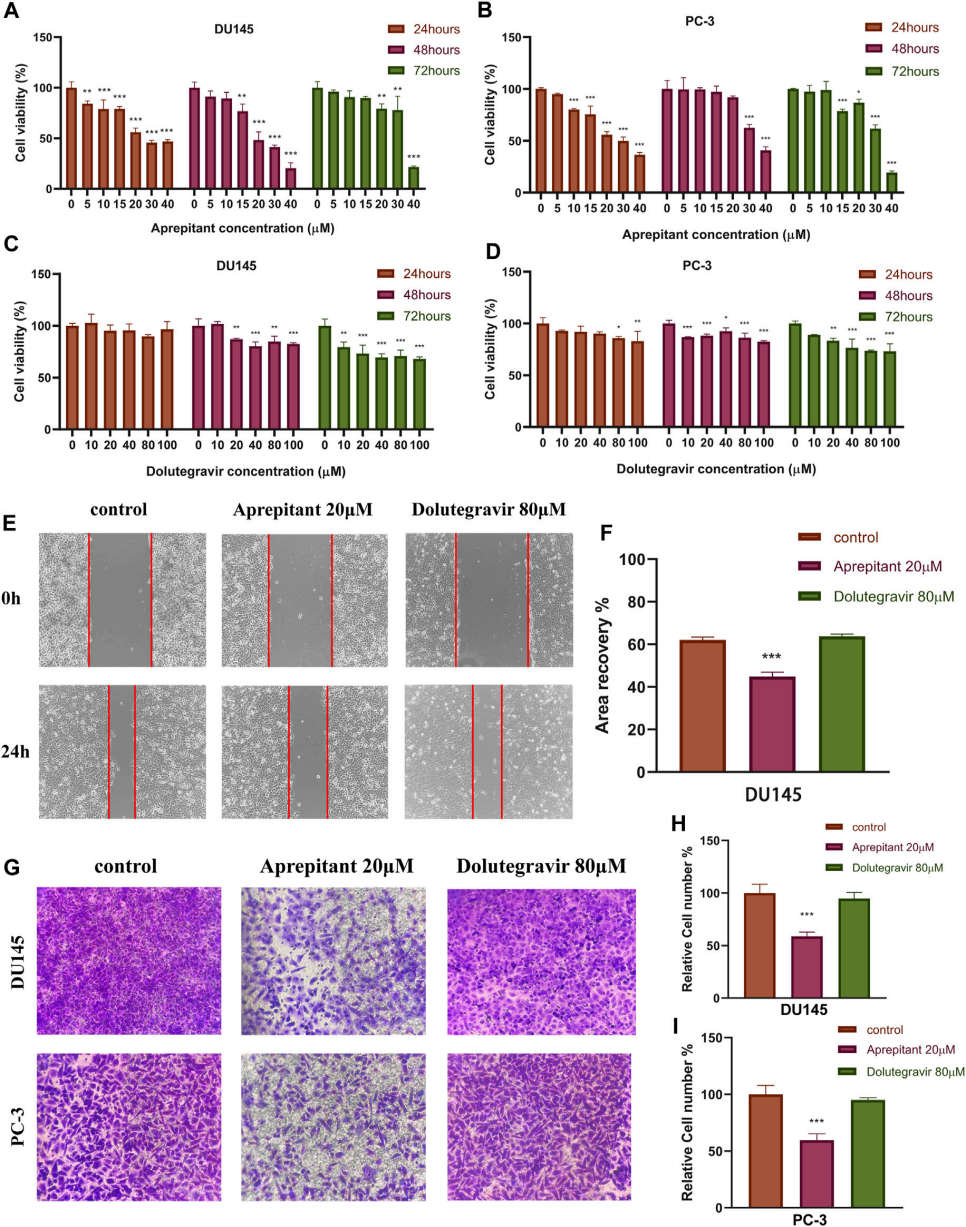
The effects of Aprepitant and Dolutegravir on the activity, migration, and invasion ability of CRPC cells were evaluated. **(A–D)** The inhibitory effects of corresponding concentrations of Aprepitant and Dolutegravir on the activity of DU145 and PC-3 cells. **(E–F)** DU145 cells were treated with 20 μM Aprepitant or 80 μM Dolutegravir to assess their effect on cell migration. **(G–I)** DU145 and PC-3 cells were treated with 20 μM Aprepitant or 80 μM Dolutegravir to observe the effect on cell invasion ability.

The CCK-8 assay was employed to assess the impact of Aprepitant and Dolutegravir on the activity of prostate cancer cells, and to evaluate their dose-dependent antiproliferative effects. The results indicate that both Aprepitant and Dolutegravir have inhibitory effects on the activity of prostate cancer cells, with Aprepitant showing a significantly superior inhibition capacity to that of Dolutegravir (Figures 9A–D). Specifically, Aprepitant exhibits robust inhibition of prostate cancer cells in the short term (24 h), but its inhibitory effect diminishes significantly over time (48–72 h) at low concentrations. Conversely, Dolutegravir exhibits no significant impact on the activity of prostate cancer cells in the short term (24 h), but its effect gradually increases over time (48–72 h). For subsequent cell migration and invasion experiments, we have selected concentrations of 20 μM for Aprepitant and 80 μM for Dolutegravir.

### Migration and invasion

We conducted wound healing and transwell assays to preliminarily evaluate the effects of Aprepitant and Dolutegravir on the migration and invasion of prostate cancer cells. Compared to the control group, the wound healing rate of DU145 cells treated with 20 μM Aprepitant was significantly reduced after 24 h (*p* < 0.001) (Figures 9E,F). However, there was no significant difference in the wound healing rate of cells treated with 80 μM Dolutegravir compared to the control group (*p* > 0.05). The transwell assay yielded similar results, as the number of cells that transversed the matrix gel was significantly reduced in DU145 and PC-3 cells treated with 20 μM Aprepitant for 24 h (*p* < 0.001), while there was no significant difference in the number of cells that crossed the matrix gel in cells treated with 80 μM Dolutegravir compared to the control group (*p* > 0.05) (Figures 9G–I).

## Discussion

Prostate cancer is the most common malignant tumor for men in Western countries and poses a severe threat to men’s health, especially in the elderly population (Siegel et al., 2019). Although ADT initially responds well to treatment, almost all patients eventually develop CRPC. In recent years, studies have found mechanisms that promote the occurrence and progression of CRPC, including androgen receptor (AR) aberrations, phosphatase and tensin homolog (PTEN) losses, DNA repair gene deletions, TP53 mutations, and RB transcriptional corepressor 1 (RB1) losses (Grasso et al., 2012; Robinson et al., 2015). However, the molecular mechanisms underlying CRPC occurrence and progression are not fully understood, and identifying novel biomarkers for CRPC diagnosis and treatment is critical. Recently, the development of machine learning algorithms has attracted researchers’ attention, and the analysis of sophisticated computer algorithms can help researchers find the critical factors of the problem from big messy data. In our study, we simultaneously adopted WGCNA and two machine learning algorithms (LASSO logistic regression and SVM-RFE) to identify two essential genes (CCNA2 and CKS2) based on the GEO database, which can be used as diagnostic biomarkers of CRPC. At the same time, we explored the biological processes, pathways, and diseases in which DEGs were enriched in primary prostate cancer and CRPC and discussed the correlation between two diagnostic biomarkers and immune cell infiltration. Finally, we employed Auto Dock Vina-based virtual screening technology to predict inhibitors targeting CCNA2 and CKS2 from FDA-approved small-molecule drugs on the market, and conducted preliminary *in vitro* experiments to explore the drug’s mechanism of action.

In our study, the GO enrichment analysis revealed that most DEGs were enriched for cell cycle-related biological functions. KEGG analysis showed that DEGs were associated with multiple metabolic pathways. GSEA showed that primary prostate cancer genes were mainly enriched in signalling-related pathways, while the CRPC gene strongly correlated with cell cycle regulation. The results of these enrichment analyses showed a significant difference between the primary prostate and CRPC in the regulation of substance metabolism and the cell cycle, which might be one of the mechanisms underlying the transformation of CRPC.

We identified two diagnostic biomarkers (CCNA2 and CKS2) through WGCNA and a machine learning algorithm. Through validation with training and testing cohorts, we found that CCNA2 and CKS2 significantly differed in expression levels between primary prostate cancer and CRPC. ROC analysis revealed that CCNA2 and CKS2 both showed strong prediction ability and could be used as diagnostic biomarkers for CRPC.

Cyclin A2 (CCNA2) belongs to a highly conserved cyclin family and promotes the G1/S and G2/M transitions by binding to and interacting with cyclin-dependent kinases 1 (CDK1) and cyclin-dependent kinases 2 (CDK2) (Pagano et al., 1992). CCNA2 is involved in the proliferation, invasion, and differentiation of normal and tumor cells. Yang et al. (2020) found that CCNA2 is associated with prostate cancer cell proliferation, invasion, metastasis, and the cell cycle (Yang et al., 2020). Gorlov et al. (2010) found that CCNA2 may be one of the main drivers of prostate cancer development (Gorlov et al., 2010). As the binding partner of the catalytic subunits of cyclin-dependent kinases (CDKs), CDC-28 protein kinase regulatory subunit 2 (CKS2) plays a regulatory role in the cell cycle process. Numerous studies have found that the abnormal expression of CKS2 is closely related to the carcinogenesis of many cancers and the enhancement of tumor proliferation, such as colorectal cancer (Yu et al., 2015), gastric cancer (Kang et al., 2009), bladder cancer (Chen et al., 2011), hepatocellular carcinoma (Ji et al., 2018), and breast cancer (Liberal et al., 2012). Mechanistically, CKS2 can support tumor cells in overriding the intra-S phase checkpoint by binding to CDK2 and producing proliferation advantages under stress conditions (Liberal et al., 2012). CKS2 can also interact with the catalytic subunit bearing CDK1, thereby activating the CDK1-Cyclin B complex (van Zon et al., 2010). The latest research has found that CKS2 can enhance mitochondrial DNA replication activity and tumor invasion by forming a complex with single-stranded DNA binding protein 1 (SSBP1) (Jonsson et al., 2019). In addition, CKS2 was found to directly induce the upregulation of cyclin A and cyclin B1 in some solid tumors (Lv et al., 2013; Shen et al., 2013). Lan et al. (2008) found that abnormal expression of CkS2 promotes tumorigenesis by inhibiting programmed cell death in prostate cancer. Interestingly, previous studies found that CCNA2 and CKS2 were highly expressed in primary prostate cancer compared to normal prostate cancer tissues. In our study, we further found that, compared with primary prostate cancer, the expression levels of CCNA2 and CKS2 in CRPC increased significantly. Prostate cancer is considered a continuous progressive disease, and as the disease progresses, CCNA2 and CKS2 increase. Therefore, we speculate that CCNA2 and CKS2 may be critical factors for the deterioration of prostate cancer, serving as new diagnostic biomarkers and therapeutic targets for CRPC.

In clinical practice, Aprepitant is commonly used to prevent chemotherapy-induced nausea and vomiting and is believed to alter cytochrome P450 activity (Patel et al., 2017). In recent years, researchers have noticed that Aprepitant can act as a neurokinin-1 receptor (NK-1R) antagonist to exert anti-tumor effects in various cancers (Muñoz and Coveñas, 2020). Mechanistically, substance P (SP) (after binding to the NK-1R) promotes mitogenesis in tumor cells, while Aprepitant (after binding to the same receptor) counteracts the SP-mediated mitogenesis and induces apoptotic mechanisms in these cells (Muñoz and Coveñas, 2013; Munoz et al., 2015). In addition to its use in preventing vomiting, Aprepitant has been found to prevent postoperative tumor recurrence and metastasis, thrombosis and thromboembolism, and is considered a smart bullet against cancer, with a large amount of preclinical and clinical data demonstrating its potential as a broad-spectrum anti-tumor drug (Muñoz and Coveñas, 2020). In prostate cancer, Ebrahimi et al. (2022) have demonstrated through *in vitro* and *in vivo* experiments that Aprepitant can inhibit cell proliferation and migration via the SP/NK-1R pathway and significantly prolong the survival time of mice (Ebrahimi et al., 2022). Interestingly, we found that Aprepitant may also act as an inhibitor of CCNA2 to exert anticancer effects. In our study, a machine learning algorithm predicted that CCNA2 may be one of the key driving factors for the transformation of primary prostate cancer to CRPC, and virtual screening predicted that Aprepitant and CCNA2 protein have strong binding ability. Therefore, we have reason to suspect that CCNA2 may be one of the targets of Aprepitant in inhibiting CRPC cells, together with the SP/NK-1R pathway. In addition, we further validated through *in vitro* experiments that Aprepitant has a significant inhibitory effect on the proliferation, migration, and invasion of CRPC cells. In conclusion, Aprepitant has the potential to be an anti-tumor drug for CRPC.

Dolutegravir is a newly approved next-generation HIV-1 integrase strand transfer inhibitor used in combination with other antiretroviral drugs for the treatment of HIV-1 infection in adolescents and adults (McCormack, 2014). Mechanistically, Dolutegravir exerts its antiviral activity by selectively targeting the integrase enzyme within the pre-integration complex of HIV-1. Specifically, it binds to two critical metal cations (Mg^2+^) located at the catalytic active site of integrase, causing displacement of the 3'-terminal deoxyadenosine of the viral cDNA strand. This binding event prevents the transfer of viral cDNA into the host DNA, thereby effectively inhibiting HIV-1 replication (Hare et al., 2011). Recently, however, it has been reported that Dolutegravir may have anticancer effects by inhibiting the expression of human endogenous retrovirus type K (HERV-K) in various cancer cells (Li et al., 2022). Our study has shown that Dolutegravir may also have anticancer effects by specifically binding to the CKS2 target in CRPC cells. Therefore, we posit that Dolutegravir is a promising candidate for an anticancer drug in the treatment of CRPC.

There are still certain limitations in our study. Firstly, the phenotype of prostate cancer can be further subdivided, but for the purpose of this study, we only distinguished between primary prostate cancer and CRPC. Secondly, additional databases and larger sample sizes are needed to validate the study findings. The results of this study were solely derived from the GEO database, and future research should validate the two biomarkers via functional experiments conducted *in vivo* and *in vitro*. Finally, the inhibitory effects of Aprepitant and Dolutegravir on CRPC can also be confirmed through other *in vivo* and *in vitro* experiments.

## Data Availability

Publicly available datasets were analyzed in this study. This data can be found here: https://www.ncbi.nlm.nih.gov/geo/ (GSE32269, GSE74367, and GSE66187).

## References

[B1] Adasme M. F. Linnemann K. L. Bolz S. N. Kaiser F. Salentin S. Haupt V. J. (2021). PLIP 2021: Expanding the scope of the protein-ligand interaction profiler to DNA and RNA. Nucleic Acids Res. 49 (W1), W530–w534. 10.1093/nar/gkab294 33950214 PMC8262720

[B2] Bogard N. Linder J. Rosenberg A. B. Seelig G. (2019). A deep neural network for predicting and engineering alternative polyadenylation. Cell 178 (1), 91–106.e23. 10.1016/j.cell.2019.04.046 31178116 PMC6599575

[B3] Cai C. Wang H. He H. H. Chen S. He L. Ma F. (2013). ERG induces androgen receptor-mediated regulation of SOX9 in prostate cancer. J. Clin. Invest. 123 (3), 1109–1122. 10.1172/jci66666 23426182 PMC3582143

[B4] Chen R. Feng C. Xu Y. (2011). Cyclin-dependent kinase-associated protein Cks2 is associated with bladder cancer progression. J. Int. Med. Res. 39 (2), 533–540. 10.1177/147323001103900222 21672358

[B5] Davies A. Conteduca V. Zoubeidi A. Beltran H. (2019). Biological evolution of castration-resistant prostate cancer. Eur. Urol. Focus 5 (2), 147–154. 10.1016/j.euf.2019.01.016 30772358

[B6] Ebrahimi S. Mirzavi F. Aghaee-Bakhtiari S. H. Hashemy S. I. (2022). SP/NK1R system regulates carcinogenesis in prostate cancer: Shedding light on the antitumoral function of aprepitant. Biochim. Biophys. Acta Mol. Cell Res. 1869 (5), 119221. 10.1016/j.bbamcr.2022.119221 35134443

[B7] Gorlov I. P. Sircar K. Zhao H. Maity S. N. Navone N. M. Gorlova O. Y. (2010). Prioritizing genes associated with prostate cancer development. BMC Cancer 10, 599. 10.1186/1471-2407-10-599 21044312 PMC2988752

[B8] Grasso C. S. Wu Y. M. Robinson D. R. Cao X. Dhanasekaran S. M. Khan A. P. (2012). The mutational landscape of lethal castration-resistant prostate cancer. Nature 487 (7406), 239–243. 10.1038/nature11125 22722839 PMC3396711

[B9] Hare S. Smith S. J. Métifiot M. Jaxa-Chamiec A. Pommier Y. Hughes S. H. (2011). Structural and functional analyses of the second-generation integrase strand transfer inhibitor dolutegravir (S/GSK1349572). Mol. Pharmacol. 80 (4), 565–572. 10.1124/mol.111.073189 21719464 PMC3187526

[B10] Harris W. P. Mostaghel E. A. Nelson P. S. Montgomery B. (2009). Androgen deprivation therapy: Progress in understanding mechanisms of resistance and optimizing androgen depletion. Nat. Clin. Pract. Urol. 6 (2), 76–85. 10.1038/ncpuro1296 19198621 PMC2981403

[B11] Huang M. L. Hung Y. H. Lee W. M. Li R. K. Jiang B. R. (2014). SVM-RFE based feature selection and Taguchi parameters optimization for multiclass SVM classifier. ScientificWorldJournal 2014, 795624. 10.1155/2014/795624 25295306 PMC4175386

[B12] Huang Y. Liu H. Zuo L. Tao A. (2020). Key genes and co-expression modules involved in asthma pathogenesis. PeerJ 8, e8456. 10.7717/peerj.8456 32117613 PMC7003696

[B13] Ji X. Xue Y. Wu Y. Feng F. Gao X. (2018). High-expressed CKS2 is associated with hepatocellular carcinoma cell proliferation through down-regulating PTEN. Pathol. Res. Pract. 214 (3), 436–441. 10.1016/j.prp.2017.12.006 29487004

[B14] Jonsson M. Fjeldbo C. S. Holm R. Stokke T. Kristensen G. B. Lyng H. (2019). Mitochondrial function of CKS2 oncoprotein links oxidative phosphorylation with cell division in chemoradioresistant cervical cancer. Neoplasia 21 (4), 353–362. 10.1016/j.neo.2019.01.002 30856376 PMC6411633

[B15] Kachroo P. Eraso J. M. Beres S. B. Olsen R. J. Zhu L. Nasser W. (2019). Integrated analysis of population genomics, transcriptomics and virulence provides novel insights into Streptococcus pyogenes pathogenesis. Nat. Genet. 51 (3), 548–559. 10.1038/s41588-018-0343-1 30778225 PMC8547240

[B16] Kang M. A. Kim J. T. Kim J. H. Kim S. Y. Kim Y. H. Yeom Y. I. (2009). Upregulation of the cycline kinase subunit CKS2 increases cell proliferation rate in gastric cancer. J. Cancer Res. Clin. Oncol. 135 (6), 761–769. 10.1007/s00432-008-0510-3 19034516 PMC12160174

[B17] Kantoff P. W. Higano C. S. Shore N. D. Berger E. R. Small E. J. Penson D. F. (2010). Sipuleucel-T immunotherapy for castration-resistant prostate cancer. N. Engl. J. Med. 363 (5), 411–422. 10.1056/NEJMoa1001294 20818862

[B48] Lan Y. Zhang Y. Wang J. Lin C. Ittmann M. M. Wang F. (2008). Aberrant expression of Cks1 and Cks2 contributes to prostate tumorigenesis by promoting proliferation and inhibiting programmed cell death. Int. J. Cancer 123 (3), 543–551. 10.1002/ijc.23548 18498131 PMC3262990

[B18] Langfelder P. Horvath S. (2008). WGCNA: an R package for weighted correlation network analysis. BMC Bioinforma. 9, 559. 10.1186/1471-2105-9-559 PMC2631488 19114008

[B19] Li J. Lin J. Lin J. R. Farris M. Robbins L. Andrada L. (2022). Dolutegravir inhibits proliferation and motility of BT-20 tumor cells through inhibition of human endogenous retrovirus type K. Cureus 14 (7), e26525. 10.7759/cureus.26525 35936147 PMC9345775

[B20] Liberal V. Martinsson-Ahlzén H. S. Liberal J. Spruck C. H. Widschwendter M. McGowan C. H. (2012). Cyclin-dependent kinase subunit (Cks) 1 or Cks2 overexpression overrides the DNA damage response barrier triggered by activated oncoproteins. Proc. Natl. Acad. Sci. U. S. A. 109 (8), 2754–2759. 10.1073/pnas.1102434108 21697511 PMC3286935

[B21] Lin X. Yang F. Zhou L. Yin P. Kong H. Xing W. (2012). A support vector machine-recursive feature elimination feature selection method based on artificial contrast variables and mutual information. J. Chromatogr. B Anal. Technol. Biomed. Life Sci. 910, 149–155. 10.1016/j.jchromb.2012.05.020 22682888

[B22] Lv M. Zhang X. Li M. Chen Q. Ye M. Liang W. (2013). miR-26a and its target CKS2 modulate cell growth and tumorigenesis of papillary thyroid carcinoma. PLoS One 8 (7), e67591. 10.1371/journal.pone.0067591 23861775 PMC3702500

[B23] Marques R. B. Dits N. F. Erkens-Schulze S. van Weerden W. M. Jenster G. (2010). Bypass mechanisms of the androgen receptor pathway in therapy-resistant prostate cancer cell models. PLoS One 5 (10), e13500. 10.1371/journal.pone.0013500 20976069 PMC2957443

[B24] McCormack P. L. (2014). Dolutegravir: A review of its use in the management of HIV-1 infection in adolescents and adults. Drugs 74 (11), 1241–1252. 10.1007/s40265-014-0256-y 25005775

[B25] Muñoz M. Coveñas R. (2013). Involvement of substance P and the NK-1 receptor in cancer progression. Peptides 48, 1–9. 10.1016/j.peptides.2013.07.024 23933301

[B26] Muñoz M. Coveñas R. (2020). The neurokinin-1 receptor antagonist aprepitant: An intelligent bullet against cancer? Cancers (Basel) 12 (9), 2682. 10.3390/cancers12092682 32962202 PMC7564414

[B27] Munoz M. Covenas R. Esteban F. Redondo M. (2015). The substance P/NK-1 receptor system: NK-1 receptor antagonists as anti-cancer drugs. J. Biosci. 40 (2), 441–463. 10.1007/s12038-015-9530-8 25963269

[B28] Pagano M. Pepperkok R. Verde F. Ansorge W. Draetta G. (1992). Cyclin A is required at two points in the human cell cycle. Embo J. 11 (3), 961–971. 10.1002/j.1460-2075.1992.tb05135.x 1312467 PMC556537

[B29] Pal S. K. Twardowski P. Sartor O. (2010). Critical appraisal of cabazitaxel in the management of advanced prostate cancer. Clin. Interv. Aging 5, 395–402. 10.2147/cia.S14570 21152241 PMC2998247

[B30] Paller C. J. Antonarakis E. S. (2011). Cabazitaxel: A novel second-line treatment for metastatic castration-resistant prostate cancer. Drug Des. Devel Ther. 5, 117–124. 10.2147/dddt.S13029 PMC3063116 21448449

[B31] Parker H. S. Leek J. T. Favorov A. V. Considine M. Xia X. Chavan S. (2014). Preserving biological heterogeneity with a permuted surrogate variable analysis for genomics batch correction. Bioinformatics 30 (19), 2757–2763. 10.1093/bioinformatics/btu375 24907368 PMC4173013

[B32] Patel P. Leeder J. S. Piquette-Miller M. Dupuis L. L. (2017). Aprepitant and fosaprepitant drug interactions: A systematic review. Br. J. Clin. Pharmacol. 83 (10), 2148–2162. 10.1111/bcp.13322 28470980 PMC5595939

[B33] Rebello R. J. Oing C. Knudsen K. E. Loeb S. Johnson D. C. Reiter R. E. (2021). Prostate cancer. Nat. Rev. Dis. Prim. 7 (1), 9. 10.1038/s41572-020-00243-0 33542230

[B34] Ritchie M. E. Phipson B. Wu D. Hu Y. Law C. W. Shi W. (2015). Limma powers differential expression analyses for RNA-sequencing and microarray studies. Nucleic Acids Res. 43 (7), e47. 10.1093/nar/gkv007 25605792 PMC4402510

[B35] Robinson D. Van Allen E. M. Wu Y. M. Schultz N. Lonigro R. J. Mosquera J. M. (2015). Integrative clinical genomics of advanced prostate cancer. Cell 161 (5), 1215–1228. 10.1016/j.cell.2015.05.001 26000489 PMC4484602

[B36] Roudier M. P. Winters B. R. Coleman I. Lam H. M. Zhang X. Coleman R. (2016). Characterizing the molecular features of ERG-positive tumors in primary and castration resistant prostate cancer. Prostate 76 (9), 810–822. 10.1002/pros.23171 26990456 PMC5589183

[B37] Shen D. Y. Zhan Y. H. Wang Q. M. Rui G. Zhang Z. M. (2013). Oncogenic potential of cyclin kinase subunit-2 in cholangiocarcinoma. Liver Int. 33 (1), 137–148. 10.1111/liv.12014 23121546

[B38] Siegel R. L. Miller K. D. Jemal A. (2019). Cancer statistics, 2019. CA Cancer J. Clin. 69 (1), 7–34. 10.3322/caac.21551 30620402

[B39] Sun S. Sprenger C. C. Vessella R. L. Haugk K. Soriano K. Mostaghel E. A. (2010). Castration resistance in human prostate cancer is conferred by a frequently occurring androgen receptor splice variant. J. Clin. Invest. 120 (8), 2715–2730. 10.1172/jci41824 20644256 PMC2912187

[B40] Tannock I. F. de Wit R. Berry W. R. Horti J. Pluzanska A. Chi K. N. (2004). Docetaxel plus prednisone or mitoxantrone plus prednisone for advanced prostate cancer. N. Engl. J. Med. 351 (15), 1502–1512. 10.1056/NEJMoa040720 15470213

[B41] Tibshirani R. (1996). Regression shrinkage and selection via the lasso. J. R. Stat. Soc. Ser. B Methodol. 58 (1), 267–288. 10.1111/j.2517-6161.1996.tb02080.x

[B42] Trott O. Olson A. J. (2010). AutoDock vina: Improving the speed and accuracy of docking with a new scoring function, efficient optimization, and multithreading. J. Comput. Chem. 31 (2), 455–461. 10.1002/jcc.21334 19499576 PMC3041641

[B43] Tshitoyan V. Dagdelen J. Weston L. Dunn A. Rong Z. Kononova O. (2019). Unsupervised word embeddings capture latent knowledge from materials science literature. Nature 571 (7763), 95–98. 10.1038/s41586-019-1335-8 31270483

[B44] van Zon W. Ogink J. ter Riet B. Medema R. H. te Riele H. Wolthuis R. M. (2010). The APC/C recruits cyclin B1-Cdk1-Cks in prometaphase before D box recognition to control mitotic exit. J. Cell Biol. 190 (4), 587–602. 10.1083/jcb.200912084 20733055 PMC2928021

[B45] Yang R. Du Y. Wang L. Chen Z. Liu X. (2020). Weighted gene co-expression network analysis identifies CCNA2 as a treatment target of prostate cancer through inhibiting cell cycle. J. Cancer 11 (5), 1203–1211. 10.7150/jca.38173 31956366 PMC6959059

[B46] Yu M. H. Luo Y. Qin S. L. Wang Z. S. Mu Y. F. Zhong M. (2015). Up-regulated CKS2 promotes tumor progression and predicts a poor prognosis in human colorectal cancer. Am. J. Cancer Res. 5 (9), 2708–2718. 26609478 PMC4633900

[B47] Zhang X. Coleman I. M. Brown L. G. True L. D. Kollath L. Lucas J. M. (2015). SRRM4 expression and the loss of REST activity may promote the emergence of the neuroendocrine phenotype in castration-resistant prostate cancer. Clin. Cancer Res. 21 (20), 4698–4708. 10.1158/1078-0432.Ccr-15-0157 26071481 PMC4609255

